# Migraine and Alcohol—Is It Really That Harmful?

**DOI:** 10.3390/nu17223620

**Published:** 2025-11-20

**Authors:** Anna Zduńska, Joanna Cegielska, Sebastian Zduński, Izabela Domitrz

**Affiliations:** 1 Department of Neurology, Faculty of Medicine and Dentistry, Medical University of Warsaw, 01-809 Warsaw, Poland; anna.zdunska@wum.edu.pl (A.Z.); izabela.domitrz@wum.edu.pl (I.D.); 2 Medical Rehabilitation Facility, National Medical Institute of the Ministry of the Interior and Administration, 02-507 Warsaw, Poland; sebastian.zdunski@cskmswia.gov.pl

**Keywords:** alcohol, alcohol-induced headache, migraine, tension-type headache, cluster headache, trigger/s of primary headaches

## Abstract

Alcohol is a widely consumed beverage worldwide, and headaches, including migraine, tension-type headache (TTH), and other primary headaches, are common in the general population. Although epidemiological studies have shown a correlation between alcohol consumption and headaches, the specific pathophysiological mechanism of this headache remains unknown. We reviewed articles deemed relevant to the issue of alcohol as a trigger for various headaches, those that discussed alcohol consumption in these patient groups, and those that addressed the pathophysiological and clinical aspects of alcohol and headache. The review concluded that alcohol affects both migraine and non-migraine headaches. Alcohol-induced headache, classified as a secondary headache, is a throbbing, bilateral headache that is exacerbated by physical activity and is precipitated by alcohol consumption. TTH can be precipitated by alcohol consumption, and patients with TTH have more alcohol-related problems than those with migraine. Cluster headaches (CH) are often triggered by alcohol, but surprisingly, many CH patients consume alcohol, even during attacks. The relationship between alcohol and migraine is complex. Numerous components of alcoholic beverages can influence pain triggering and are responsible for migraine attacks. Red wine is one of the most frequently cited triggers for migraine attacks, a finding not always confirmed by the few prospective studies. However, there is no safe dose of alcohol, and therefore avoidance should be recommended.

## 1. Introduction

Migraine is a common neurological disease with a chronic course and unclear pathogenesis. It affects approximately 14% of the world’s population. The prevalence of migraine is significantly higher in women and generally increases with age, reaching a peak in the age group of 40–44 years and then decreasing in older people [1].

Alcohol is widely consumed in the world, with about 2.3 billion people drinking almost 35 billion liters of pure ethanol per year [2]. It is important to remember that although alcohol in various concentrations and forms is often a socially acceptable recreational drink, it is both an intoxicating drug and an addictive substance. Its harmful side is reflected in the fact that approximately 3 million people worldwide die each year directly from alcohol intoxication or from the organ and traumatic effects of its consumption [3].

Although epidemiological studies have shown a correlation between alcohol consumption and headaches, the specific pathophysiological mechanism of this headache remains unidentified [4].

## 2. Materials and Methods

This review presents the results of peer-reviewed articles published over the past twenty-five years on the association of alcohol with headache, and specifically migraine. For this purpose, a literature search was conducted in PubMed and Medline using the following terms: “headache and alcohol,” “migraine and alcohol,” “cluster headache and alcohol,” “tension-type headache and alcohol,” and “alcohol-hangover headache,” as well as “headache and wine,” “migraine and wine,” and “lifestyle and headache”.

A combined search was conducted, limiting the publications from 2000 to 10 May 2025. Additional searches for articles were conducted by hand searching bibliographies, previous peer-reviews, and by using Google Scholar references. The main searches were conducted from 10 February 2025 to 10 May 2025. Inclusion criteria were as follows: (1) only articles in English; (2) all types of articles: observational studies, cross-sectional studies, clinical trials, case-control studies; (3) only human studies; (4) literature based only on the adult population.

After screening 167 potentially relevant publications, 102 articles were included in the review. As a result of additional searches and analyses, 61 additional papers were included. Ultimately, the authors decided to include and cite 163 articles that were most closely related to the topic of the current review.

Articles considered relevant to the issue of alcohol as a trigger for various types of headaches, including migraine, TTH, and CH, as well as articles discussing alcohol consumption in these patient groups, were included. In addition, literature was selected that addressed the pathophysiological and clinical issues of the relationship between alcohol and headache.

## 3. Alcohol Effects on Non-Migraine Headache

### 3.1. Alcohol-Induced Headache

According to the International Classification of Headache Disorders, 3rd (ICHD-3), alcohol-induced headache (AIH) is included as secondary headache and classified in the section ‘Headache attributed to a substance or its withdrawal’ [5]. ICHD-3 identifies two types of secondary headaches directly related to alcohol: immediate and delayed [6]. Immediate AIH (IAIH) develops within 3 h of alcohol consumption and subsides within 72 h after cessation of alcohol. Symptoms occurring after 5–12 h are called delayed AIH (DAIH). In both cases, the headache is pulsating, bilateral, and worsens with physical activity [6]. DAIH, which occurs in the morning after drinking alcohol, when the blood alcohol level drops and reaches zero, is one of the most common, secondary headaches. DAIH can occur in a person who does not have a specific primary headache type [7], but migraineurs are at increased risk of developing it compared to those without migraine [8]. Phenotypically, both types of headaches are similar, which can make it somewhat difficult to diagnose [9]. Mostofsky et al. indicate that 1–2 drinks of alcohol do not correlate with headache, but five or more drinks are associated with an increased risk of its occurrence [10]. AIH is more common in light to moderate drinkers than in regular drinkers. Alcohol-related headaches may be accompanied by other symptoms such as loss of appetite, tremor, dizziness, nausea, tachycardia, irritability, and decreased concentration [11]. Darker alcoholic beverages such as red wine, whiskey, and bourbon contain congeners, which are natural byproducts of alcoholic fermentation and are more likely to cause AIH than clear alcoholic beverages such as gin or vodka [12]. The exact mechanism of AIH is unknown, but it may involve alcohol’s action on intracranial blood vessels, altered sleep patterns, or an inflammatory mechanism via altered cytokine pathways and prostaglandin release. Alcohol-induced magnesium deficiency may also have pathophysiological significance for AIH [12]. Ethanol is known to induce delayed hypersensitivity of the trigeminal nerve within 4–6 h of administration. Maxwell et al. showed that a similar effect is exerted by non-toxic acetate formed from acetaldehyde (an ethanol metabolism product), which is in fact responsible for the suggested AIH [13]. Patients prone to AIH should limit alcohol consumption and ensure good hydration [11].

### 3.2. Tension-Type Headache

According to the ICHD-3, tension-type headache (TTH) is a disease in which recurrent headache episodes last from 30 min to 7 days. This type of headache is characterized by bilateral location, a pressure or tension sensation (without throbbing), mild to moderate intensity, and is not aggravated by physical activity. It occurs without nausea or vomiting but may be accompanied by photophobia or phonophobia (but never both). In chronic TTH (CTTH), the headache occurs on average ≥15 days per month for >3 months, in episodic form (ETTH)—for less than 15 days a month [6].

In a single study by Panconesi et al., involving 47 patients with TTH, a history of alcohol consumption revealed that 46.8% were abstainers, 36.1% consumed alcohol occasionally, and 17% used it regularly. The percentage values of the subgroups of patients with TTH were similar to those of patients with migraine. None of the patients with TTH reported alcohol as a trigger [14]. However, most studies have shown that patients with TTH consumed more alcohol than migraine patients or those without headaches [15,16,17,18]. At the same time, there was no association between alcohol consumption and the risk of TTH or unclassified headache [18,19].

Most studies on alcohol as a trigger for TTH also include migraine patients. Few studies have shown, that alcohol as a trigger factor occurs in a similar percentage of patients with TTH and migraine (30% vs. 40%) [20,21]. Other studies have shown that alcohol rarely triggers migraine and TTH [14,19,22,23]. Table 1 presents a summary of studies covering the issue of alcoholic beverages (AD) as triggers of TTH.

### 3.3. Cluster Headache

Cluster headache (CH) is an extremely debilitating disorder with trigeminal-vegetative symptoms. The pain occurs unilaterally and is located in the area of innervation of the first branch of the trigeminal nerve, with accompanying autonomic symptoms, and clustering of symptoms during an attack that lasts 15–180 min [24]. In episodic CH (ECH), ailments occur in periods lasting from 7 days to 1 year, separated by pain-free periods lasting at least 1 month, and is referred to as. In chronic form (CCH), attacks occur for more than 1 year without remission, or with remission periods lasting less than 1 month [6].

In various studies, alcohol was reported as a trigger for CH attacks by 37.3% [25] to 55.0% of patients [26]. The most frequently mentioned alcoholic drink in this context was beer. They were indicated by 57% of patients. Red wine and spirits/hard alcohol were noted as triggers in approximately 50% of patients [27].

Lund et al. assessed the lifestyle of 400 patients with CH. Unhealthy lifestyle factors were more commonly reported by patients with CH than in the control group. Although significantly fewer patients than the control group reported alcohol consumption, the prevalence of unhealthy alcohol consumption was comparable in both groups. However, patients with CH were more likely to consume alcohol in a harmful manner. Alcohol consumption was more common in ECH than in CCH, but at the same time, fewer ECH patients than CCH ones consumed alcohol in an unhealthy or harmful way. It was surprising that as many as 14.3% of patients consumed alcohol during attacks, even though it is a widely known and recognized cause of CH attacks [28]. This may be explained only by the general perception of alcohol as a sleep aid, and sleep was disturbed in CH patients, compared to the control group (sleep efficiency was lower, and sleep latency and REM sleep latency were longer) [29].

In a study by Steinberg et al. of 500 CH patients, 28.1% of them never or rarely consumed alcohol, 29.1% consumed 1–2 standard units per week, 33.8% consumed 3–4 standard units per week, and 8.9% consumed 1–2 standard units per day. A significantly higher percentage of ECH patients (35.9%) than those with CCH (18.2%) regularly consumed alcohol, with consumption ranging from three to four standard units per week. A total of 74.5% of CH patients consumed alcohol [26]. Similarly, the study by Sjöstrand et al. confirms increased alcohol consumption in CH patients [30]. However, it should be emphasized that in reality, most CH patients avoid alcohol consumption during the cluster period [27,31]. At the same time, patients suffering from CHs were more likely to have suffered head injuries than migraine sufferers. They were more likely to be responsible for them, probably due to specific lifestyle behaviors, including excessive alcohol consumption [32].

At the same time, compared to the general population, CH patients consume less alcohol; therefore, CH appears to protect against hazardous alcohol consumption. Furthermore, predictors of hazardous alcohol consumption in CH patients are similar to those in the general population [33].

## 4. Alcohol and Migraine

According to the ICHD-3, migraine is a recurrent headache with attacks lasting 4 to 72 h. Typically, the headache is unilateral, pulsating, and moderate to severe in intensity. It is exacerbated by physical activity and is accompanied by nausea and/or photophobia and phonophobia. Chronic migraine is a headache occurring 15 or more days per month, with typical migraine symptoms occurring on at least 8 days per month; the headache persists for more than 3 months [6].

Migraine sufferers are at increased risk of AIH. In some patients, alcohol can trigger a migraine attack within a few hours of consumption, typically within about three hours. This should then be considered a typical AIH, although distinguishing alcohol-induced migraine from AIH can be difficult, due to, among other factors, the increased sensitivity of migraineurs to very small amounts of alcohol [7].

### 4.1. Potential Mechanisms

There are no studies on alcohol and headache that specifically indicate which compounds contained in various alcoholic beverages are responsible for causing headaches [34].

#### 4.1.1. Ethanol

Ethanol is a likely trigger for migraine, and a frequently suggested mechanism is its vasodilatory effects. Ethanol directly induces stimulation of meningeal nociceptors, dilates meningeal blood vessels via the release of calcitonin gene-related peptide (CGRP) and endothelial nitric oxide (NO), and induces neurogenic inflammation in the trigeminovascular system [35,36]. Ethanol, even at low concentrations, has been shown to release CGRP [36]. The importance of CGRP in the pathophysiology of migraine is strongly supported by numerous research results. It has been shown that: (1) CGRP levels are elevated in migraineurs during and between migraine attacks, (2) CGRP is decreased after migraine abortive and prophylactic treatment, (3) CGRP administration can induce migraine-like headaches in migraineurs, and (4) CGRP antagonist drugs and anti-CGRP monoclonal antibodies are effective in the treatment of migraine [37]. Studies show the effect of ethanol on headaches and the induction of neuroinflammation through its molecular action on toll-like receptor 4 (TLR4) and transient receptor potential vanilloid 1 (TRPV1) [38]. TRPV1 plays an important role in the modulation of trigeminal sensory processing [39]. Ethanol upregulates TRPV1, also known as the capsaicin/heat receptor and the vanilloid receptor, which is a confirmed pain trigger and TLR4 expression level; both receptors trigger a neuroinflammation response that promotes AIH manifestation [38]. Following TRPV1 activation and upregulation and activation of TLR4 in trigeminal nerves, inflammatory factors (e.g., CGRP, substance P) are transduced, which may be a major mechanism of AIH [40]. TRPV1 is highly expressed in small and medium-sized peripheral ganglia neurons, approximately 10–20% of trigeminal ganglia (TG) neurons being TRPV1-positive. Furthermore, 70% of CGRP-positive neurons have been shown to colocalize with TRPV1-positive neurons in the trigeminal nucleus, similar to trigeminal nerve fibers innervating the dura mater [41]. TRPV1 is also present in the endings of central nociceptive fibers, although its function at this level remains poorly understood [42]. Studies suggest that ethanol promotes serotonin (5-HT) release, stimulates 5-HT reabsorption by platelets, and induces analgesia. Among various types of alcoholic beverages, red wine induces the greatest 5-HT release [38]—see the section below.

In turn, acetaldehyde, a metabolite of ethanol, is a known agonist of trantient receptor potential ankyrin 1 (TRPA1). The TRPA1 ion channel transduces oxidative stress and triggers neurogenic inflammation. Moreover, high doses of alcohol activate microglia, significantly increasing the production of reactive oxygen species (ROS). Oxidative stress, transduced by the TRPA1 ion channel on C fibers, may initiate the release of CGRP and neurogenic inflammation associated with the pathophysiology of migraine [43]. Ethanol can also induce or increase oxidative stress by producing ROS and reactive nitrogen species (RNS) [44]. It is possible that the cutaneous allodynia reported during migraine attacks is induced by ethanol via systemic production of acetaldehyde, which, via the release of CGRP, engages the CGRP receptor on Schwann cells [45]. It is oxidative stress that may be the common denominator linking ethanol with migraine [43]. Ethanol may also cause headaches through its metabolite—acetate, which increases the formation of extracellular adenosine and thus causes pain by stimulating adenosine A2A receptors [46].

**Figure 1:**
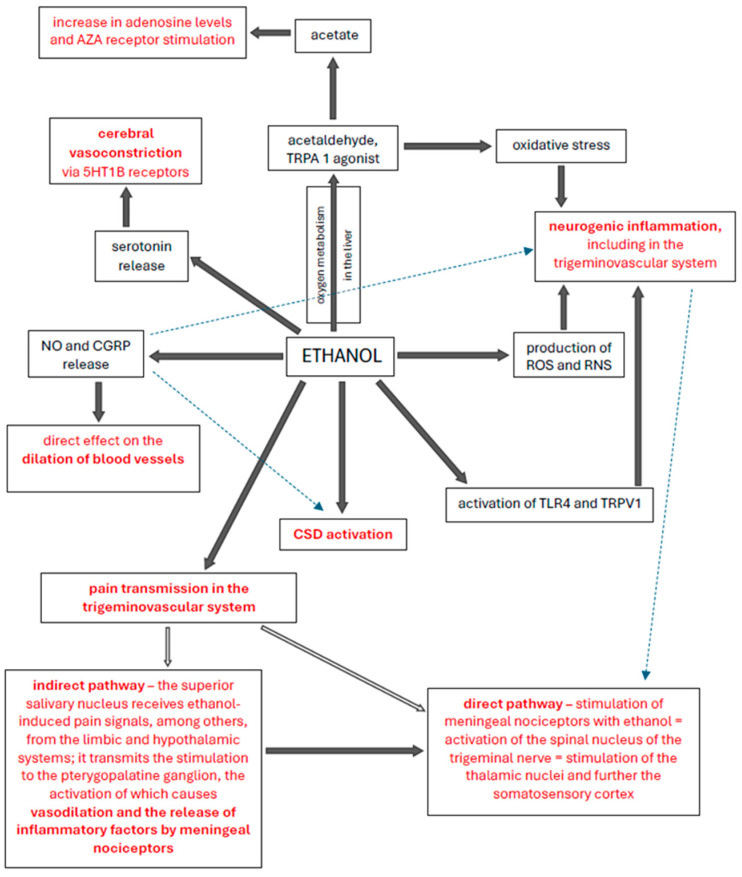
Multidirectional influence of ethanol on the mechanisms of headache triggering. Abbreviations: ROS (reactive oxygen species), RNS (reactive nitrogen species), NO (nitric oxide), CSD (cortical spreading depression), CGRP (calcitonin gene-related peptide), TRPA1 (transient receptor potential ankyrin 1), TRPV1 (transient receptor potential vanilloid 1), TLR4 (toll-like receptor 4).

One hypothesis is that ethanol induces cortical spreading depression (CSD), which is believed to be an important mechanism underlying migraine. CSD is defined as an electrophysiological phenomenon affecting neurotransmitters, ionic balance, and cerebral blood flow [47]. Alcohol consumption has a dose-dependent effect on CSD, with acute alcohol consumption slowing CSD, while chronic alcohol consumption accelerates this process. Acute alcohol consumption affects the function of the Na^+^/K^+^ ATPase, resulting in free radical production, while chronic alcohol consumption negatively affects the function of antioxidant enzymes. Furthermore, ethanol impairs astrocyte function and prolongs the duration of headache attacks [48]. CSD is associated with increased ROS levels, and increased ROS production is associated with chronic alcohol consumption [49]. Acute ethanol consumption has a depressant effect on the central nervous system, and at the cerebral cortex level, alcohol reduces cortical excitability or promotes the activity of cortical inhibitory circuits, likely by increasing gamma-aminobutyric acid neurotransmission. Ethanol is known to dilate cerebral dura mater vessels. However, alcohol-induced vasodilation does not explain all primary headaches or AIH. A common pathogenic mechanism at the cortical or subcortical/brainstem level seems more likely [5]. The most plausible theory seems to be the induction of AIH by neurogenic inflammation [35,36]. The multidirectional effects of ethanol on the mechanisms of headache induction described in the article are illustrated in Figure 1.

#### 4.1.2. Wine and Other Alcoholic Beverages

A headache caused by red wine usually occurs within 30 min to 3 h of consumption. Wine does not have to be consumed in excessive amounts to cause a headache—one or two glasses are sufficient, which is not a large dose of ethanol. Therefore, ethanol does not seem to be the main cause of headaches in this case [50]. Wine contains, however some components, such as biogenic amines, sulphites, nitrites, flavonoid phenols, which substances may be associated with migraine headaches [4]. Histamine is involved in the pathophysiology of migraine and may be a factor responsible for wine-induced headache. Tyramine content in both red and white wine is trace [50]. Wine and some AD have been observed to inhibit platelet function and are associated with reduced platelet aggregation [51], in other studies this association was observed only in red wine [52]. It is suspected that flavonoids and the most common biogenic amines (BA) found in wine are associated with migraine: serotonin, histamine and tyramine [4].

##### Polyphenols

Polyphenols are phytochemical compounds associated with the health-promoting properties of red wine, although they constitute only 0.1% of its composition. Over 100 polyphenols have been identified in red wine, and their presence depends on grape ripening conditions (sun exposure, geographic location, soil type), the vinification process itself, including fermentation and aging conditions, and the yeast strains used in winemaking. Phenolic compounds have a significant impact on wine quality, color, and flavor [53]. Maceration processes during red wine vinification facilitate the extraction and diffusion of polyphenols into the juice, resulting in red wine having a polyphenol concentration up to 10 times higher than in white wines [54].

The total amount of polyphenols in wine has been estimated to range from 800 to 6000 mg/L for red wines and from 50 to 350 mg/L for white wines. These compounds are directly related to the quality of wines and are responsible for most of their antioxidant activity [55]. The potential of polyphenols as free radical scavengers or antioxidants is predicted based on their chemical activity. Various mechanisms have been proposed to explain their antioxidant properties. First, they can eliminate free radicals and ROS by donating a hydrogen atom, but they can also act through a process called single electron transfer. The second mechanism is based on their ability to form complexes with metal ions such as Fe^2+^ and Cu^2+^, thus reducing one of the factors contributing to free radical production. Third, they can activate antioxidant enzymes [56]. The bioavailability of polyphenols and the resulting biological activity are largely dependent on the processes of intestinal digestion, hepatic absorption, conjugation and elimination, and metabolism by microflora, which vary among individuals [57].

Some classes of polyphenols are found primarily in wine (stilbenes, anthocyanins) and others only in beer (chalcones and flavanones), while flavanols and flavan-3-ols occur in similar concentrations in both beverages. Polyphenols also play a key role in beer quality, as they influence transportation and storage time, flavor stability, clarity, and color [54].

Polyphenols are generally divided into two main classes: flavonoids and non-flavonoids, which include resveratrol [54].

Resveratrol gained notoriety in the early 1990s following the publication of an epi-ideological study known as the “French paradox,” which addressed the observation that, despite high levels of dietary saturated fat and cigarette smoking in France, mortality rates from coronary heart disease were low. This has been attributed to moderate consumption of red wine and its polyphenol content, including resveratrol [58]. Resveratrol reduces acetaldehyde generation and increases acetaldehyde metabolism to acetic acid by increasing acetaldehyde dehydrogenase 2 (ALDH2) in cultured human peripheral lymphocytes [59]. Resveratrol is a powerful antioxidant found in few foods, with grapes and red wine being its richest sources [53]. The average concentration of total resveratrol in red wine is 7 mg/L, in rosé wine 2 mg/L, and in white wine 0.5 mg/L [60]. However, significant differences in its content have been found in red and white wines across different countries [59]. The minimum daily intake for resveratrol benefits is estimated at 1 g per day, and it is relatively safe at doses up to 5 g. However, the required daily intake cannot be achieved through wine or any other food [58]. Resveratrol is currently being widely researched for its anti-aging, neuroprotective, anti-inflammatory, and antioxidant properties, and for its use in medicine [60]. Despite its significant beneficial biological effects on human health, resveratrol is characterized by a poor pharmacokinetic profile due to its low aqueous solubility, poor chemical stability during digestion, and low bioavailability [60]. The resveratrol content is not comparable in wine and beer, as these compounds are absent or present in beer at very low concentrations. This may be explained by the fact that resveratrol is found in hops, and a small amount of hops is typically added during beer production [54].

Resveratrol and flavonoid compounds have shown a positive effect on the prevention of coronary heart disease and other cardioprotective effects by changing the lipid profile, reducing insulin resistance, increasing the bioactivity of nitric oxide (NO) and lowering blood pressure and glucose levels [59].

Flavonoid phenolic compounds are probably the most likely cause of red wine headache in both migraineurs and non-migraineurs [13]. Phenols are a substrate for the enzyme phenolsulfotransferase (PST), which occurs in two forms: PST-M, which inactivates monoamines (tyramine and dopamine), and PST-P, which degrades phenol and *p*-cresol in the intestine. Researched that extracts from red wine contain very strong PST inhibitors, especially numerous flavonoids, which strongly and specifically inhibit PST-P. About 30% of the flavonoid fraction are catechins and anthocyanins (responsible for the color of red wine), which are strong PST-P inhibitors in vitro [50]. The presence of flavonoid radicals is responsible for the ability of red wine to induce migraine and possibly headache attacks in non-migraineurs [13]. In turn, a deficit of PST-P has been identified in dietary migraine. Other studies have shown that in migraineurs, the activity of PST-M, responsible for inactivation of dopamine and 5-HT, was reduced, but not the activity of PST-P, without a significant difference in dietary migraine [50].

##### Biogenic Amines

Consuming foods and beverages containing BA is safe for most people because the human body is able to metabolize them using monoamine oxidases (MAO) and diaminoxidases (DAO). However, in some cases, the human body’s detoxification mechanism is not sufficiently effective, and excessive levels of BA in consumed foods and beverages are harmful. A dose of 100 mg/L or 100 mg/kg of BA is considered safe for most people, but for AD, this limit is much lower because ethanol may reduce the effectiveness of the detoxification mechanism [61].

BAs can be formed at various stages of wine production. Some are components of grapes, and they can also be produced by yeast during alcoholic fermentation and by the action of bacteria involved in malolactic fermentation [62]. Among the BAs, histamine is the most important, not only because it is the most toxic but also because ethanol and other amines (e.g., tyramine, phenylethylamine, tryptamine) increase its toxicity by inhibiting enzymes (DAO and MAO) involved in histamine detoxification in humans [62].

A large variability in BA amounts generally occurs between white and red wines due to maceration with grape skins, which occurs only in red wine production, and the high fermentation temperature, which is short-lived or absent in white wine production. The most common sources of BA in beer are malt, barley, the fermentation itself, and contaminating microflora [63]. However, very little data have been published on BAs levels in spirits.

Serotonin

5-HT in wine is produced during malolactic and alcoholic fermentation under the influence of yeast and lactic acid bacteria [64]. It is formed by the decarboxylation of L-tryptophan. 5-HT is present in wine at insufficient concentrations, ranging from pg to ng/mL. Further research is needed to identify and quantify 5-HT in wine and other ADs [65].

As previously mentioned, red wine causes the greatest 5-HT release compared to other AD. Various species of red wine contain varying concentrations of flavonoids, and the increased release of 5-HT is induced only by flavonoids with a molecular weight of 500 Da or greater. In addition, 5-HT inhibitors are present in some wines [66]. Red wine is a potent releaser of 5-HT from platelets and also strongly inhibits the binding of 5-HT to 5-HT1 receptors. Therefore, release of 5-HT from central stocks/depots may be a likely mechanism for wine-induced headache [50]. It has also been suggested that oral mast cells may promote headache by releasing 5-HT, prostaglandins, and histamine [67]. Red wine has been shown to have endothelium-dependent vasorelaxant activity, probably via a NO-mediated pathway [68]. Moreover, wine inhibits 5-HT and noradrenaline reuptake as well as MAO activity through its polyphenolic component resveratrol and action on 5-HT receptors [13].

##### Histamine

Histamine releases NO from the endothelium, and it has been suggested that NO release from cerebral blood vessels, perivascular nerve endings, or brain tissue is an important molecular trigger of spontaneous headache [68]. Histamine can activate TRPV1 and also promote the expression of TLR4 receptors, triggering a neuroinflammatory response that promotes the manifestation of AIH, as previously mentioned [69]. Some studies have shown that wines high in histamine increased AIH more than wines low in histamine, while others have found no such correlation [70].

Histamine intolerance (HIT) is a term used to describe a type of food intolerance that is a set of adverse reactions resulting from the accumulation or ingestion of histamine, with a very wide range of clinical symptoms. This is most often accompanied by reduced activity of intestinal DAO [71]. DAO is a copper-dependent amino acid oxidase encoded by the AOC1 gene located on chromosome 7 (7q34–36). In the gastrointestinal tract, DAO is the main enzyme responsible for the degradation of histamine ingested with food or produced by the intestinal microbiota [72]. Some bacterial strains (Lactobacillus casei TISTR 389 and Lactobacillus bulgaricus TISTR 895) possess an enzyme that ensures endogenous histamine synthesis in the human body [73].

HIT may be caused by genetically determined reduced DAO activity associated with single nucleotide polymorphisms (SNPs) in the AOC1 gene. Currently, the most significant SNPs in the AOC1 gene associated with predisposition to HIT are: c.47C>T (rs10156191), c.995C>T (rs1049742), and c.1990C>G (rs10449793) [73]. DAO is found mainly in the small intestine; therefore, inflammatory conditions in the gastrointestinal mucosa (e.g., Crohn’s disease, ulcerative colitis, celiac disease, intestinal dysbiosis, parasitic infections) may result in impaired DAO activity [61]. Moreover, histamine metabolism may be disturbed by taking drugs—DAO inhibitors (a large group of drugs, including acetylsalicylic acid, naproxen, ibuprofen often used in the acute treatment of migraine, or amitriptyline used in prophylactic treatment) [71].

One of the key factors contributing to impaired histamine metabolism is alcohol consumption. Alcohol itself may contain histamine, and it also reduces the level of the DAO enzyme and stimulates the synthesis of endogenous histamine [72].

HIT symptoms can involve multiple body systems, presenting with a wide range of nonspecific gastrointestinal and extraintestinal symptoms, including headaches. Symptoms range in severity from mild to severe [74].

The treatment of HIT is a low-histamine diet, which primarily involves avoiding foods containing excessive amounts of histamine and BAs. These foods include seafood, aged cheese and sausages, fermented soy products, chocolate, avocado, nuts, milk, legumes, and fruits such as bananas, yeast, and alcohol. Patients who respond to the diet should continue it for a month until symptoms subside. Foods are then gradually reintroduced [72]. Once histamine tolerance levels are established, long-term tolerance should be periodically reassessed, as factors such as medications, microbiome changes, small intestine health, alcohol consumption, and menstruation can alter tolerance levels [73]. DAO inhibitor medications should be discontinued. DAO supplementation may also be used, as it aids in the degradation of ingested histamine and enhances the effects of the diet [72]. In addition to a low-histamine diet and/or DAO dietary supplementation, antihistamines can be used in acute and clinically more severe cases [73].

Furthermore, studies have shown that red wines may increase blood histamine levels to a greater extent than other AD [75]. It contain significantly more than twice the concentration of BAs, including histamine, than white wines and other AD [76,77].

After the administration of DAO, a reduction in the symptoms of histamine intolerance, including headaches [78], a significant reduction in headaches in patients with migraine, and a decrease in the percentage of patients taking triptans were observed [79]. At the same time, selected genotypes and allelic variants of DAO associated with reduced enzyme activity were shown to be associated with an increased risk of migraine, especially in women [80]. A high incidence of DAO deficiency has been observed in migraine patients [81]. The histamine content in AD, especially red wine, and possibly concomitantly consumed foods rich in histamine, e.g., mature cheeses, may play a key role in triggering migraines and other headaches [4]. Currently, the main way to prevent or reduce symptoms associated with histamine intolerance due to DAO deficiency, including headaches, is to follow a low-histamine diet [74]. A low-histamine diet effectively reduces intolerance symptoms, including headaches, and potentially increases serum DAO [82].

The European Food Safety Authority has proposed a potential reference dose for histamine of 50 mg in healthy individuals, but below the detectable limits for individuals with HIT. According to the Food and Drug Administration, the tolerable level of histamine is 50 mg/kg. For wine, various countries have set upper limits ranging from 2 mg/L (Germany) to 10 mg/L (Australia and Switzerland) [63].

**Figure 2:**
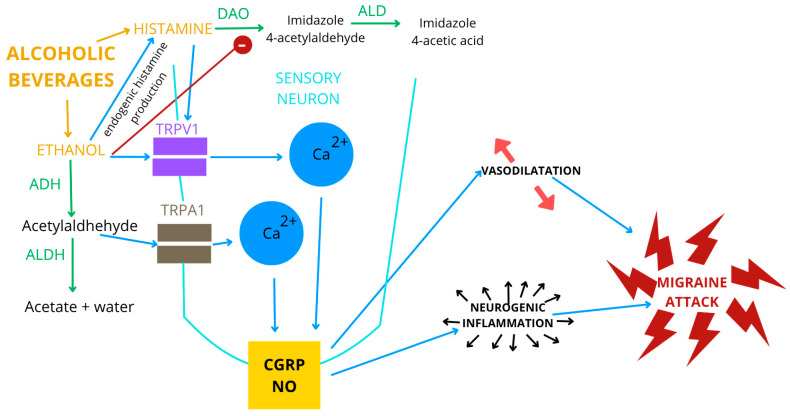
Mapping of beverage components to pathways involved in alcohol metabolism, and their association with neuroinflammation, vascular effects, and migraine attack provocation. Abbreviations: DAO (diamine oxidase), ALDH (aldehyde dehydrogenase), ADH (alcohol dehydro-genase), NO (nitric oxide), CGRP (calcitonin gene-related peptide), ALD (aldehyde dehydrogenase), TRPV1 (trantient receptor potential vanilloid member 1), TRPA1 (trantient receptor potential ankyrin 1). The green factors are potentiating an enzymatic reaction. The red factors are stimulating an enzymatic reaction. The blue factors are stimulating calcium channels and the release of signaling molecules. TRPA1 and TRPV1 are both calcium-permeable, nonselective cation channel found in sensory neurons. ADH and ALDH play a critical role in ethanol breakdown, with the key intermediate product acetaldehyde, which is a TRPA1 agonist, while ethanol is a TRPV1 agonist. Sensory stimulation and downstream signaling: activation of TRPA1 and TRPV1 leads to the release of NO and CGRP. NO and CGRP are key signaling molecules involved in neurogenic inflammation and vasodilation—processes associated with migraine attack generation. Alcoholic beverages contain both ethanol and histamine. Ethanol itself reduces the level of the enzyme DAO (involved in histamine breakdown) and stimulates endogenous histamine synthesis. Histamine can activate TRPV1 via Ca^2+^ influx. Adapted according to [43,45,69,71,72,83].

Figure 2 shows the mapping of beverage components to pathways involved in alcohol metabolism, and their association with neuroinflammation, vascular effects, and migraine attack provocation.

##### Tyramine

Tyramine is a naturally occurring BA derived from the amino acid tyrosine. It has been proposed that migraine patients with some food sensitivity may have a genetic deficiency of the enzyme responsible for breaking down tyramine. One study demonstrated migraine provocation after administration of 100 mg tyramine to patients with diet-induced migraine, but other studies have not confirmed this association [84]. Furthermore, it has been shown that the tyramine content in both red and white wine is low [50].

The European Food Safety Authority has proposed potential reference doses for tyramine of 600 mg for healthy individuals and less than 6 mg for individuals taking classic MAO inhibitors [63].

##### Sulphites

Sulphites occur naturally in many foods and beverages through fermentation, for example in beer and wine, but have also been used as a food additive for many years, due to their antiseptic, antioxidant, and antimicrobial properties. However, excessive sulphite consumption can cause symptoms such as headaches, nausea, stomach irritation, and respiratory problems, especially in people with asthma [85]. Since 2005, sulphites have been included in the list of allergens, making labeling of wines with sulphite levels greater than 10 mg/L mandatory in the European Union (EU). The acceptable daily intake of sulphites, according to the World Health Organization (WHO), is 0.7 mg/kg body weight [86]. Due to the risk to human health, the total sulphite content, expressed as sulphur dioxide, must not exceed 150 and 200 mg/L for conventional red and white wines, respectively, and 100 and 150 mg/L for organic red and white wines, respectively [55].

Sulphites, which are used to preserve wine, have not been convincingly shown to trigger migraine headaches [50]. Sulphites are much more common preservatives in other foods, such as dried fruit, which do not cause headache attacks [13]. It appears that these substances may cause headaches indirectly, by inducing histamine release from mast cells, as symptoms have been observed to occur only during periods of increased histamine sensitivity [87].

##### Nitrites/Nitrates

Nitrates and nitrites are widely distributed in the environment, and their main source in the human diet is vegetables. Furthermore, these compounds are used as additives to improve food quality and protect against microbiological contamination and chemical changes. Processed (cured) meat is another source of nitrates and nitrites in our diet. Nitrites (sodium nitrite—E249, potassium nitrite—E250) and nitrates (sodium nitrate—E251, potassium nitrate—E252) are approved as food additives in the EU [88]. The contribution of drinking water to nitrate intake is typically low (less than 14%). However, due to the use of inorganic fertilizers, nitrate levels in water resources have increased [89]. After nitrate intake, approximately 60–70% is readily absorbed and rapidly excreted in urine. About 25% is secreted in saliva, of which about 20% is converted to nitrite by commensal bacteria on the tongue (using bacterial nitrate reductase). Endogenous nitrates and nitrites are produced in the L-arginine/NO-synthase pathway and are the end product of NO oxidation [88]. Up to 70% of systemic NO is generated by endothelial NO-synthase (eNOS), while neuronal NOS (nNOS) is present in neurons of the central and peripheral nervous system. These enzymes synthesize NO from the amino acid l-arginine and molecular oxygen to achieve vasodilation, blood pressure regulation, inhibition of endothelial inflammatory cell recruitment, and platelet aggregation [90]. Released NO is a highly reactive compound, so excess NO is rapidly oxidized (in the blood) to nitrite and nitrate by oxyhemoglobin or oxymyoglobin proteins [88]. Polyphenols found in food can increase NO production from nitrite in the gastrointestinal tract and also protect NO from oxidative damage (prolonging its half-life) [90].

As with all essential nutrients, excessive intake of nitrates and nitrites is associated with an increased risk of negative health effects. The WHO has established an acceptable daily intake for nitrate ion at 3.7 mg/kg body weight and for nitrite ion at 0.06 mg/kg body weight [90]. Nitrates are unstable in an acidic environment and spontaneously decompose into nitrites and nitrogen dioxide. Nitrites formed during nitrate metabolism, as well as those obtained from food, can additionally react in the gastrointestinal tract with precursors of *N*-nitroso compounds (such as amines and amides) and result in the formation of *N*-nitroso compounds. The reaction of nitrites with secondary amines is considered particularly dangerous, as it leads to the formation of carcinogenic nitrosamines [88].

Nitrogen occurs naturally in grape juice because the grapevine obtains nitrogen from nitrates, ammonia, or urea used during cultivation and is commonly referred to as yeast-fed nitrogen [91]. However, complete fermentation is often necessary to replenish nitrogen sources and achieve optimal concentrations. The wine industry commonly uses inorganic nitrogen sources, such as diammonium phosphate, to achieve the required nitrogen level. It has been reported that an initial yeast-fed nitrogen concentration of 250 mg/L in grapes is sufficient for efficient fermentation, but when it is less than 140 mg/L, nitrogen supplementation is important to avoid slow or stalled fermentation [92]. Nitrates are also present in beer; in addition to water, nitrate sources can include barley, malt adjuncts, and hops [93]. However, other foods contain more nitrates than wine and beer, as mentioned above [84].

NO can lead to vascular dilatation and headache stimulation in susceptible individuals [94]. However, because NO has a short half-life, it is very unlikely that NO from food will ever reach the central nervous system [95]. The results of studies evaluating blood nitrite/nitrate levels in migraine patients are conflicting. Some studies have shown higher nitrite/nitrate levels in migraine patients [96], others—lower in migraine with aura [97] and in migraine compared with control [98], and still other studies found no difference between migraine and control [99,100].

Typical ranges of the discussed ingredients for individual beverages are summarized in Table 2.

### 4.2. Alcohol as Migraine Trigger

In a systematic review of the literature published between 1958 and 2015, including 12,400 participants, 21% of patients identified alcohol as a trigger for migraine [106].

Onderwater et al. conducted a study of 2197 patients, of whom 35.6% reported alcohol as a trigger for migraine. Of the migraine sufferers who consumed alcohol, 42.5% reported alcohol as a trigger. Most often (77.8%), it was red wine, although it triggered an attack in only 8.8% of the study participants. At the same time, patients reporting alcohol as a trigger for migraine were more likely to have migraine without aura and a more severe course of migraine. More than 25% of migraine patients did not consume alcohol because of its possible triggering effect [107]. Similarly, in other retrospective studies, AD were reported as a migraine trigger in approximately 20–40% of migraine patients [21,108,109,110,111,112,113]. In studies conducted in India, Japan, Turkey and Italy, the percentage of alcohol or wine as a factor provoking migraine attacks was very low and amounted 0–6% [14,19,22,114,115,116,117,118], and in China low—10% [23], which is probably related to the alcohol habits of their inhabitants. Besides cultural differences, the estimated prevalence of this factor as a migraine trigger varies significantly depending on the research approach and the population studied [5].

In the study by Hauge et al., 59% of patients reported that alcohol triggered a migraine attack within 1 h of exposure, but only 14% reported migraine headache the next day. To prevent attacks, 91% of patients in the group who experienced an alcohol-induced reaction stated that they did not drink any alcohol or avoided some types of alcohol [113]. Table 3 lists AD as a triggering factor for migraine attacks.

Many studies have found no significant differences in the frequency of alcohol-triggered migraine attacks in MO and MA [108], although patients reported alcohol as a trigger for MO, not MA [14,112]. A higher susceptibility to red wine and a lower percentage of alcohol sensitivity were reported in MA [19,22]. No differences in susceptibility to alcohol were observed between men and women [108].

In Europe, the results vary by country, with red wine being most frequently reported in the UK and white wine in France and Italy. This may be due to differences in the popularity and availability of particular drinks in these countries [50].

Headaches with triggers were associated with higher pain intensity, greater headache-related disability, and medication ineffectiveness compared to headaches without triggers [119].

There are few prospective studies evaluating alcohol as a risk factor for migraine attacks and its consumption among migraine patients. One of them is the PAMINA study conducted by Wöber et al., which showed that the most popular AD was red wine, consumed by 75.8% of migraine patients. At the same time, alcohol had no adverse effect on the provocation of migraine attacks, and beer consumption in the days preceding the onset of headache reduced the risk of headache and migraine [120]. Similarly, a prospective study by Leone et al. found alcohol to be a trigger factor in 12% of migraine patients, but prospectively only in 4% of migraine attacks [121]. In the study by Casanova et al., in a group of 1125 patients diagnosed with migraine who completed an electronic diary for 90 days, it was shown that alcohol was associated more with reduce risk of an attack than being a trigger of a migraine attack (6.7% vs. 0.9%), which suggests that strict recommendations to avoid it may not be justified [122]. In the studies, wine is the most frequently reported avoided trigger (30%) [123]. As we can see, in numerous retrospective studies alcohol, especially red wine, is often mentioned as a factor provoking migraine attacks, in fact, prospective studies report a limited importance of alcohol as a migraine trigger [5].

The overall quality of evidence regarding alcohol as a trigger for migraine attacks is low, as most studies are cross-sectional studies or patient surveys, traditionally based on information from paper diaries. Only some studies [10,119] use electronic diaries, which reduce recall bias and improve treatment adherence [124]. There are only a few high-quality, randomized, controlled trials investigating dietary factors as triggers for migraine attacks [10,119].

Dietary studies that attempt to link specific foods, including alcohol, to migraine are limited by the lack of placebo controls, which makes them difficult to use. It should be noted that alcohol is a contributor rather than a cause of migraine attacks [125]. Alcohol is one of the most commonly cited triggers for migraine, but population and clinical studies suggest that migraine patients are less likely to drink, and alcohol has not been associated with migraine in diary studies [7].

Furthermore, comparison of studies is limited because some of them focused on one specific trigger, such as alcohol, whereas others assessed a variety of triggers, including alcohol. In some studies, patients were provided with a list of triggers, while in others, patients self-reported triggers [124].

The heterogeneity of ADs, combined with differences in their ability to trigger migraine, increases the complexity of the relationship between alcohol and migraine [126]. Due to the heterogeneity of available ADs, these results are not easy to interpret [84].

### 4.3. Alcohol Consumption in Migraine Patients

There are several large-scale epidemiological studies that show that alcohol consumption is lower among people suffering from headaches. The prevalence of headaches among people who consume alcohol has been studied in various settings [34].

The association of alcohol consumption with migraine has varied across several cross-sectional studies. A study conducted in a Dutch population of 5176 migraine patients showed that migraine patients consumed AD less frequently than the general population, especially in patients with chronic migraine (CM) [127]. Similarly, other studies have shown that migraineurs consumed less alcohol [17,18,123,128,129,130,131,132,133,134,135,136,137]. At the same time, patients with active migraine were more likely to report low alcohol consumption compared to those with a history of migraine, suggesting that alcohol consumption patterns may be driven by current pain experiences. Alcohol consumption is lower among people experiencing active migraine, while those who do not experience active migraine may no longer avoid alcohol [135,138,139]. Similarly, patients with frequent migraine attacks were more likely to be in the lowest alcohol consumption category [135], those with less frequent migraine consumed more alcohol [140], and those with episodic migraine (EM) consumed alcohol less frequently than those with CM [16]. Most cross-sectional studies have shown an inverse association between migraine frequency and alcohol consumption, which probably reflects the fact that migraine sufferers avoid AD [141]. Some studies have shown similar alcohol consumption in patients with and without migraine [117], and several studies have shown a higher rate of hazardous alcohol consumption in patients with migraine [117,142,143,144]. In the study by Takeshima et al., the simple index of habitual drinking was significantly higher in women suffering from migraine [19].

No significant differences in the risk of high alcohol consumption were found between MA and MO [132,135].

In the Panconesi et al. study involving 401 migraine patients, 50.1% were abstainers, 32.1% were occasional alcohol users, and 17.7% were regular alcohol consumers [14].

Alcohol consumption in the Japanese is strongly inhibited by lack of activity ALDH2, because people with ALDH2 deficiency are more susceptible to alcohol-induced flushing and hangover reactions. People with ALDH2 deficiency and headaches, including migraine, consumed alcohol statistically significantly less often than those with properly functioning ALDH2, and additionally, patients with migraine consumed alcohol less often than patients with TTH [145].

Some studies focused on assessing alcohol consumption among patients with migraine [16], others in a broader group of patients with neurological conditions, including migraine [137], and still others assessed alcohol consumption additionally, focusing on other factors co-occurring with migraine, e.g., the occurrence of anemia [144], depression [139], cognitive impairment [138], nausea and vomiting as leading symptoms [133]. Table 4 summarizes studies assessing alcohol consumption in patients with migraine.

Alcohol consumption and migraine are inversely correlated. The exact mechanism underlying this observation may indicate that migraine leads to alcohol avoidance [146].

### 4.4. Alcohol Consumption and the Risk of Migraine

Alcohol consumption does not affect the risk of migraine after adjusting for age and gender (logistic analysis) [18,19]. Occasional and regular drinkers are at even lower risk of CM and medication overuse headache (MOH) than abstainers [14]. Increased dietary alcohol consumption was associated with a reduced risk of migraine or severe headaches in one study, particularly in older adults, suggesting that alcohol-induced migraine may not be as serious as previously thought [2]. Also, a twin study found that drinking alcohol once a week or once a month was associated with a reduced risk of migraine [132]. In the developed multifactorial model for predicting individual migraine attacks, no association was found between alcohol consumption and migraine attacks, although the model was developed based on data from only 178 migraine patients [147]. Similarly, in the Head-Hunt study involving 6209 patients with migraine, significant trends were observed towards a decrease in the frequency of migraine with increasing alcohol consumption [136]. However, one should not be too quick to conclude a causal relationship, as migraine patients may self-limit their alcohol consumption [84]. In turn, a prospective study by Vives-Mestres et al. focused on the role of alcohol as a potential trigger for migraine attacks within 24–48 h after alcohol consumption. No significant effect on the probability of a migraine attack within 24 h after alcohol consumption was found, while the probability of a migraine attack was even slightly lower as a result of alcohol consumption 24 to 48 h [140]. On the other hand, Mostofsky et al. showed a higher risk of next-day migraine headache after consuming 5 or more drinks of alcohol compared to non-alcoholics, which was related to the amount of alcohol consumed [10].

### 4.5. Risk of Alcohol Use Disorders in Patients with Migraine

Alcohol use disorders (AUD) are characterized by impaired ability to control alcohol consumption and compulsive alcohol use over a prolonged period of time. A recent meta-analysis showed positive risk associations between alcohol consumption and the occurrence of AUD and mortality. Even at an average intake of 20 g/day, the risk of developing AUD is almost three times higher than in nondrinkers, and the risk of death from AUD is about twice as high as in nondrinkers [148].

A Canadian study of 36,984 people found no difference in the incidence of alcohol dependence over a 12-month period between those with and without migraine [149]. Subramaniam et al. assessed lifetime AUD (i.e., abuse or dependence) among adults with various chronic pain conditions, including migraine, in Singapore. Compared with those without migraine, those with migraine had a higher risk of lifetime AUD [136]. In another study, patients with migraine were less likely to have alcohol-related problems (Alcohol Use Disorders Identification Test) than patients with TTH [150]. Most epidemiological studies have not shown that migraine is associated with a statistically increased risk of AUD in the general population, although the results are not entirely conclusive [151]. The increased risk of AUD in patients with primary headaches may be due to the effect of alcohol in triggering headache attacks in some susceptible individuals. Headache patients often believe that alcohol precipitates at least some of their attacks. The considerable heterogeneity in studies reporting alcohol consumption in headache patients limits detailed comparisons between them [151].

### 4.6. Genetic Predisposition

Yuan et al. conducted a Mendelian randomization study to assess the causal association of alcohol consumption with the risk of migraine. Independent SNPs associated with potential risk factors in large genome-wide association studies were selected as instrumental variables. Mendelian randomization analyses showed an association of genetically predicted increased alcohol consumption with a lower risk of migraine and an inverse effect of genetic susceptibility to migraine on reduced alcohol consumption [152]. Heavy drinkers tend to have a higher tolerance for headaches and metabolize alcohol more quickly, and therefore are less exposed to toxic byproducts (i.e., acetaldehyde) that can trigger migraines [153]. In addition, headache patients abstain from alcohol because it is often a trigger for headache attacks. In this context, genetic polymorphisms of alcohol-metabolizing enzymes, alcohol dehydrogenase (ADH) and ALDH, have been shown to be associated with the risk of triggering a migraine attack after alcohol consumption [46]. Variants in three genes encoding alcohol-metabolizing enzymes, the ALDH2 gene and two ADH genes (ADH1B and ADH1C), have been associated with varying risks of developing certain alcohol-related diseases. These variants are more common in Asian populations [154]. ADH1B accelerates ethanol oxidation and its conversion to acetaldehyde, whereas ALDH2 is involved in acetaldehyde detoxification. The high-activity ADH1B*2 variant (Arg47His mutation) and the ALDH2*2 polymorphism, resulting in markedly reduced enzymatic activity, are common among East Asians. These variants promote acetaldehyde accumulation, leading to a phenotype characterized by facial flushing and discomfort after alcohol consumption. Genetic polymorphisms in the ALDH2 and ADH1B genes significantly influence alcohol metabolism and the accumulation of toxic metabolites, thus determining the risk of various alcohol-related diseases [155]. The ADH2 His allelic variant may be associated with the risk of triggering migraine attacks after alcohol consumption in migraine patients, while the ADH2 Arg/His genotype should be associated with a reduced risk of migraine [46].

Evidence suggests that oxidative stress is associated with migraine pathophysiology and that genetic variation may influence individual oxidative capacity. Papasavva et al. conducted an association analysis of the studied SNPs associated with oxidative stress and migraine triggers. They demonstrated statistically significant associations of selected variants for the haplotype of glutathione S-transferase Pi 1 (GSTP1), which affects brain cell detoxification, with alcohol consumption as a trigger for migraine attacks [145].

### 4.7. Recommendations for Migraine Patients Regarding Alcohol Use

By the early 21st century, numerous epidemiological studies had established that moderate alcohol consumption, especially wine, was associated with a lower risk of cardiovascular disease. It was also clear that these benefits could be enhanced by red wine consumption due to the additional effects of polyphenols, which have been shown to have beneficial properties independent of the presence of alcohol [156]. However, more recent data contradict this assumption and, on the contrary, indicate an increased risk of cardiovascular disease even at low levels of consumption. In fact, the WHO and the European Society of Cardiology clearly state that there is no safe level of alcohol consumption for cardiovascular health [157,158]. The relationship between cardiovascular disease and alcohol consumption is multifaceted and complex [51].

Migraine, especially with aura and occurring frequently, increases the risk of certain cardiovascular diseases. People with migraine who have frequent attacks consume AD less frequently than control patients and those with less frequent attacks. Therefore, the uncritical recommendation to abstain from alcohol in all migraineurs is under discussion. Before concluding that alcohol is responsible for a migraine attack, patients should carefully record their alcohol consumption to check the amount of alcohol consumed, the specific types of drinks, the frequency of headaches, and any situations or stresses present before alcohol consumption that may generate a migraine attack [7].

All migraine patients should be advised to keep a headache diary, which can often uncover unrecognized triggers that can be treated. It can also help to understand them that some factors perceived as triggers are not actually triggers. Newer headache diary applications that identify triggers and collaterals using mathematical modeling can enable patients to better understand their headaches and show individual triggers [125]. Anticipating migraine attacks can lead to preventive treatment, provide patients with the ability to plan for upcoming attacks, and reduce interictal anxiety [159]. In patients who have an excessive fear of headache between attacks, ‘cephalgyaphobia’ should be recognized and treated. The presence of cephalgyaphobia in episodic migraine is an important predictor of the transformation of migraine into a chronic form and of medication overuse [160]. Patients should be advised to focus on a healthy lifestyle rather than trying to avoid potential migraine triggers. Maintaining a healthy weight, getting enough sleep, and exercising regularly are protective factors against chronic migraine. However, restrictions on certain dietary factors, such as excessive alcohol consumption, should also be recommended [125].

Alcohol is a psychoactive substance that leads to many health problems, such as road accidents and cancer; it directly causes impairment of attention, cognitive functions and dexterity, as well as aggressive behavior and loss of control over emotions. Alcohol problems occur at every age, but in the age group of 25–49 years, alcohol has the greatest impact on cancer mortality and life disability [146]. The main causes of alcohol-related premature death burden are liver cirrhosis, road traffic accidents and tuberculosis [161]. Globally, 4.1% of all new cancer cases in 2020 were attributed to alcohol consumption, with ¾ of cases occurring in men. More than 100,000 cancer cases in 2020 were caused by light to moderate drinking of about one to two alcoholic drinks per day [162]. Alcohol consumption is a well-established cancer risk factor and is associated with cancers of the mouth and throat, esophagus, liver, colon, and breast [163].

WHO states that there is no safe dose of alcohol. At the same time, some authors suggest that low alcohol consumption is not a contraindication in patients with headache. However, each patient makes individual decisions based on their own experience. Migraineurs may gain unforeseen health benefits by avoiding alcohol consumption, e.g., avoiding the negative effects of alcohol consumption, discussed above [146].

Formulating general recommendations regarding alcohol consumption in migraineurs is complicated by individual variability resulting from genetic, metabolic, and lifestyle differences. Factors such as genetic predisposition to alcohol metabolism, general diet, and the presence of other medical conditions may influence the individual effects of alcohol/red wine. Attack frequency and the presence of co-triggering factors should also be considered. Patients with high-frequency migraine and suspected histamine intolerance should be advised to discontinue alcohol consumption. However, in patients with low-frequency migraine, testing the type/dose of beverage with diary-based monitoring may be recommended, bearing in mind that, according to the WHO, there is no safe dose of alcohol, as shown in the algorithm below.
high-frequency migraine attack
trial of low-histamine dietwith suspected histamine
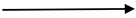
+intolerance
alcohol avoidance







type/dose beveragelow-frequency migraine attack
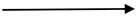
+

diary-based monitoring

## 5. Limitations

Our study has several limitations that should be noted. First, the scope of our study may be limited by the exclusion of articles published in languages other than English. Second, we may have missed useful studies indexed in other databases. Furthermore, potential bias may result from a lack of systematic rigor in the search methodology and the variety of article types included.

## 6. Conclusions

Alcohol affects both migraine and non-migraine headaches. Regarding non-migraine headaches, it’s important to emphasize that:

- AIH, classified as a secondary headache, is a pulsating, bilateral headache that worsens with physical activity and is provoked by alcohol consumption.
- TTH can be provoked by alcohol consumption, and some studies have reported that alcohol consumption by patients with TTH is similar or greater to that of migraineurs, and even TTH patients have more alcohol-related problems than migraineurs.
- CH is often provoked by alcohol, but strangely enough, many CH patients consume alcohol, even during attacks.

The relationship between alcohol and migraine remains the subject of much research. It is important to emphasize that:

- There are no studies clearly indicating which alcohol components are responsible for migraine attacks; the following are taken into account: ethanol and components found in red wine, such as flavonoid phenols, serotonin, histamine, tyramine, sulfites and nitrites.
- Alcohol, especially red wine, is one of the most frequently mentioned factors provoking migraine attacks, which is not always confirmed by the few prospective studies.
- Migraineurs, especially those with active migraine, frequent attacks, and chronic migraine, avoid drinking alcohol.
- Alcohol consumption does not seem to affect the risk of migraine, and the risk of AUD is not increased in migraineurs.
- Further research is needed to assess the influence of genetic factors on the association between migraine and alcohol.
- Alcohol leads to many health problems, including some cancers and road accidents, and the WHO states that there is no safe dose of alcohol, so all patients, including those with migraine, should be advised to avoid drinking alcohol.

The key question of the biological mechanism by which alcohol precipitates migraine attacks remains unanswered. It can be assumed that triggers are able to destabilize subcortical structures that are an integral part of homeostasis, activating genetically sensitized pathways involved in migraine headache. Understanding how this happens may help to better understand the pathophysiology of migraine [111].

## Figures and Tables

**Table 1:** Summary of clinical studies on alcohol as a trigger for TTH attacks. Abbreviations: TTH (tension-type headache), CTTH (Chronic TTH), ETTH (Episodic TTH), M (migraine), MA (migraine with aura), MO (migraine without aura).

Reference	Country	Method of Study	Patients No	Diagnosis	% of Patients Reporting Alcohol As a Trigger	Trigger Factor
Spierings 2001 [20]	USA	Questionnaire	17/38	TTH/M	29/42	Alcohol
Wöber 2006 [21]	Austria	Cross-sectional study	22/66	TTH/MO	31.8/40.9	Alcohol
Takeshima 2004 [19]	Japan	Questionnare	1125/122/ 301/41	ETTH/CTTH/ MO/MA	1.9/0.0/ 1.4/0.0	Wine
Karli 2005 [22]	Turkey	Questionnaire	31/23/33	ETTH/MA/MO	6.5/0/6.1	Alcohol
Panconesi 2013 [14]	Italy	Questionnaire	47/401	TH/M	0/4.9	Alcohol
Wang 2013 [23]	China	Cross-sectional survey	344/394	TH/M	7.6/11.4	Alcohol

**Table 2:** Summary of nutrients contained in alcoholic beverages. Abbreviations: LOD (limit of detection).

Nutrients	Alcoholic Beverages				References
	Red Wine	White Wine	Beer	Spirits	
Polyphenols [mg/100 mL]	108.38	59.91	71.48	1.25	[101]
Flavonoids [mg/100 mL]	82.5	34.83	45.9	0	[101]
Resveratrol [mg/100 mL]	1.83	0.59	0	0	[101]
Serotonin [µg/l]	<LOD − 2.28		No data available	No data available	[64]
Histamine [mg/L]	<LOD − 28.1	<LOD – 16.6	0.1–5.0	No data available	[61,102,103]
Tyramine [mg/L]	0.5–37.5	0–6.8	0.1–58.3	No data available	[104,105]
Nitrates [mg/L]	-		0.21–54.1	-	[89,93]
Nitrites [mg/serving]	-		< LOD	-	
Nitrosamines [μg/serving]	0.019 μg/136 g		0.53 μg/357 g	0.02 μg/41 g	

**Table 3:** Summary of clinical studies on alcohol as a trigger for migraine attacks in migraine sufferers. Abbreviations: MA (migraine with aura), MO (migraine without aura).

Reference	Country	Method of Study	Patients No	Diagnosis	% of Patients Reporting Alcohol As a Trigger	Type of Alcohol
Onderwater 2019 [107]	The Netherlands	Cross-sectional questionnaire study	2197	MO	35.6	Alcohol
					77.8	Red wine
Wöber 2006 [21]	Austria	Cross-sectional study	66	MO	40.9	Alcohol
Kelman 2007 [108]	USA	Observational retrospective questionnaire study	1750	MO	37.8	Alcohol
Fukui 2008 [109]	Brazil	Observational retrospective questionnaire study	200	MO	34	Alcohol
					1.5	Red wine
					10.5	White wine
					1.5	Soft drink
Andress-Rothrock 2010 [110]	USA	Questionnaire survey	200	MO	20.5	Alcohol
Finocchi 2012 [111]	Italy	Observational retrospective questionnaire study	100	MO	20	Wine
Hauge 2010 [112]	Denmark	Observational retrospective questionnaire study	347	MA	34.5	Alcohol
Hauge 2011 [113]	Denmark	Questionnaire survey	126	MA	22 20.91 11.5 9.41 5.23 18	Alcohol Red wine Liquor Champagne or sparkling wine White wine Beer
Yadav 2010 [114]	India	Observational retrospectivequestionnaire study	182	MO	0	Alcohol
Sulena 2020 [115]	India	Observational retrospective questionnaire study	1065/180	MO/MA	2.9	Food items including alcohol
Takeshima 2004 [19]	Japan	Questionnaire survey	213/31	MO/MA	1.4/0	Wine
Karli 2005 [22]	Turkey	Observational retrospective questionnaire study	33/23	MO/MA	6.1/0	Alcohol
Mollaoğlu 2013 [116]	Turkey	Prospective cohort study	126	MO	3.9	Alcohol
Özcan 2019 [117]	Turkey	Questionnaire	142	MO + MA	2	Wine
Panconesi 2013 [14]	Italy	Cross-sectional study	401	MO + MA	4.9	Alcohol
					2.9	Wine

**Table 4:** Summary of clinical studies investigating alcohol consumption in migraine patients. Abbreviations: MA (migraine with aura), MO (migraine without aura), AD (alcoholic drink), mth (month).

Reference	Country	Method of Study	Migraine Patients No	Control Group (No Headache) No	% Migraine Drinkers	% Control Group Drinkers	Type of Alcohol and/or Method of Consumption
Van den Hoek 2024 [127]	The Netherlands	Questionnaire	5176	8370	63	78	Current alcohol consumption
Lebedeva 2016 [15]	International	Interview	496	1014	26	27.8	Light alcoholic drinks
					9.5	9.3	Strong alcoholic drinks
Zlotnik 2014 [128]	Israel	Questionnaire	95	597	78.95	81.41	Alcohol
Lisicki 2021 [123]	International	Questionnaire	59	77	71 40 37 2	75 33 35 8	Wine consumption Once a month Once a week Everyday
Schramm 2021 [129]	Germany	Questionnaire	584 MA 168 MO 416	634	13.7 11.8	13.6	Alcohol abuse
Schramm 2015 [130]	Germany	Questionnaire	724	1074	0.3	1	Alcohol use (daily or almost daily drinking of AD)
Hagen 2018 [18]	Norway	Questionnaire	644	12,815	9.8	7	Never
					65.5	51.2	<4/mth
					17.6	15.8	4–7/mth
					3.6	6.5	>8/mth
Luo 2021 [131]	USA	Questionnaire	517	2683	10.02	13.61	Had any alcohol in the last 24 h
Le 2011 [132]	Denmark	Questionnaire	8044 MA-3086 MO-4958	23,821	27.7 (MA-29.8, MO-26.3)	18.9	Alcohol
					21.8 (MA-21.0, MO-22.4)	20.5	Never/seldom Monthly
					50.5 (MA-49.2, MO-51.3)	60.6	Weekly
Kim 2016 [133]	Korea	Cross-sectional study	51	102	5.9	22.5	Alcohol drinking once a week
Scher 2005 [134]	Netherland	Questionnaire	620 MA-192 MO-396	5135	MA-48, MO-51 MA-26, MO-24 MA-23, MO-23 MA-3.3, MO-2.4	37 23 32 7.3	0 drinks/day <1 drinks/day, 1–3 drinks/day >4 drinks/day
Rist 2015 [135]	USA	Cross-sectional study	7042	25,755	1.17 1.19 1.13 1.22	39.3 80.4 59.1 78.1	Total alcohol consumption Beer White wine Red wine
Aamodt 2006 [136]	Norway	A population-based cross-sectional study	6209	13,873	35	31.6	Abstainers
					36.5	34.6	1–4 glasses/2 weeks
					16.7	19.8	4–14 glasses/2 weeks
					1.5	2.2	>14 glasses/2 weeks
Geisler 2021 [137]	Germany	Questionnaire	135	612	1.1	2.56	Alcohol consumption as a % of energy requirement

## Data Availability

Not applicable—the article is a review of previously published research.
